# The potential utility of integrated reflectance confocal microscopy-optical coherence tomography for guiding triage and therapy of basal cell carcinomas

**DOI:** 10.7150/jca.47026

**Published:** 2020-08-18

**Authors:** Nicusor Iftimia, Aditi Sahu, Miguel Cordova, Gopi Maguluri, Melissa Gill, Christi Alessi-Fox, Salvador Gonzalez, Cristian Navarrete-Dechent, Ashfaq Marghoob, Chih-Shan J. Chen, Milind Rajadhyaksha

**Affiliations:** 1 Physical Sciences, Inc., 20 New England Business Ctr. Drive, Andover, MA, USA.; 2 Dermatology Service, Department of Medicine, Memorial Sloan Kettering Cancer Center, New York, NY, USA.; 3 Department of Pathology, SUNY Downstate Medical Center, Brooklyn, NY, USA.; 4 Caliber Imaging and Diagnostics, Rochester, NY, USA.; 5 Alcala University, Madrid, Spain.; 6 Pontificia Universidad Catolica De Chile, Santiago, Chile.

**Keywords:** basal cell carcinomas, invasive non-surgical treatments, reflectance confocal microscopy, optical coherence tomography

## Abstract

The increasing rate of incidence and prevalence of basal cell carcinomas (BCCs) worldwide, combined with the morbidity associated with conventional surgical treatment has led to the development and use of alternative minimally invasive non-surgical treatments. Biopsy and pathology are used to guide BCC diagnosis and assess margins and subtypes, which then guide the decision and choice of surgical or non-surgical treatment. However, alternatively, a noninvasive optical approach based on combined reflectance confocal microscopy (RCM) and optical coherence tomography (OCT) imaging may be used. Optical imaging may be used to guide diagnosis and margin assessment at the bedside, and potentially facilitate non-surgical management, along with long-term monitoring of treatment response. Noninvasive imaging may also complement minimally invasive treatments and help further reduce morbidity. In this paper, we highlight the current state of an integrated RCM/OCT imaging approach for diagnosis and triage of BCCs, as well as for assessing margins, which therefore may be ultimately used for guiding therapy.

## Introduction

Approximately 2.4 million new cases of basal cell carcinomas (BCCs) are diagnosed every year in the U.S. and about another 800,000 in other regions of the world 1-3. BCCs occur most commonly in older (> 65 years) and in middle-aged people (40-65 years), but, recently its incidence has been increasing in younger people, too 4,5. Approximately 80-90% of the cases occur in the head-and-neck area, including 65% on the face, which includes in high-risk functional anatomical areas such as the nose, eyes, ears or mouth 6,7. Although mostly non-fatal, BCCs can cause large-scale anatomical destruction, resulting in morbidity, physical disfigurement, loss of function (breathing, hearing, swallowing and vision) and psychological trauma. Preserving quality of life - preserving tissue and anatomy, cosmesis and function, reducing morbidity and pain, minimizing collateral damage - is a key objective during treatment. BCCs are conventionally treated with either surgical excision with wide margin control or Mohs micrographic surgery with microscopic margin control 8,9. Both surgical treatments produce high cure rates 10,11. However, the increasing rates of incidence and prevalence of BCCs worldwide has led to the development and use of minimally invasive non-surgical treatments, which offer lower morbidity and improved quality of life. Non-surgical treatments include topical therapy (Imiquimod), photodynamic therapy, radiotherapy, and laser ablation and/or coagulation 12-17. Guiding the decision and choice of an appropriate minimally invasive therapy is dependent on determining BCC depth and subtype 18,19.

Low-risk non-aggressive shallow (depth < 400 µm) superficial and early nodular types of BCC are amenable to non-surgical treatments, whereas deeper (depth > 400 µm) nodular and high-risk micronodular, infiltrative and sclerosing subtypes require treatment with surgical excision or Mohs surgery. Current diagnosis, depth and subtyping are determined with biopsy followed by histopathological analysis. Instead of biopsy, alternatively, a noninvasive imaging approach may be used to guide diagnosis and determine depth and subtype. Noninvasive imaging may also complement minimally invasive treatments and help further reduce morbidity and preserve quality of life.

We investigated the use of a non-invasive optical imaging method, based on integrating reflectance confocal microscopy (RCM) and optical coherence tomography (OCT) into a single instrument. Many studies have shown that both OCT and RCM imaging, when used individually and independently can noninvasively detect superficial and nodular BCCs with sensitivities and specificities in the range 80-95% and 70-90%, respectively 20-26. Since RCM provides cellular-level resolution, it can be used to accurately detect the morphological features of BCCs and provide high diagnostic accuracy. However, imaging depth is limited to ~200 µm, and therefore determination of margins is possible only for superficial tumors in the papillary dermis. On the other hand, OCT imaging reaches deeper, into the reticular dermis to depth of ~1.5 mm and can be used to detect deeper nodular, micronodular, infiltrative and sclerosing tumors and delineate deeper margins. Therefore, the combined use of RCM and OCT within the same instrument provides enhanced capabilities for skin cancer detection and especially for therapy guidance. Their sequential use on a specific lesion is not desirable since spatial co-registration of the images enables enhanced cancer detection and margins delineation. Furthermore, sequential use doubles the imaging time and thus the costs of the procedure. In addition, it requires the acquisition of two instruments, which are more expensive than a combined instrument. Our approach to integrate RCM and OCT within a single instrument was thus a logical step toward further advancing the translation and clinical use for diagnosis of BCC and margin delineation.

In this paper, we highlight the unique feature the integrated RCM/OCT instruments by summarizing the results of a preliminary patient study. In this study, an OCT/RCM instrument with a hand-held probe was tested for BCC diagnosis and delineation of deep and lateral margins. Based on spreading depth measurements, triage of BCCs into deeper aggressive high-risk or shallower non-aggressive low-risk types was possible.

## Material and Methods

**Figure 1:**
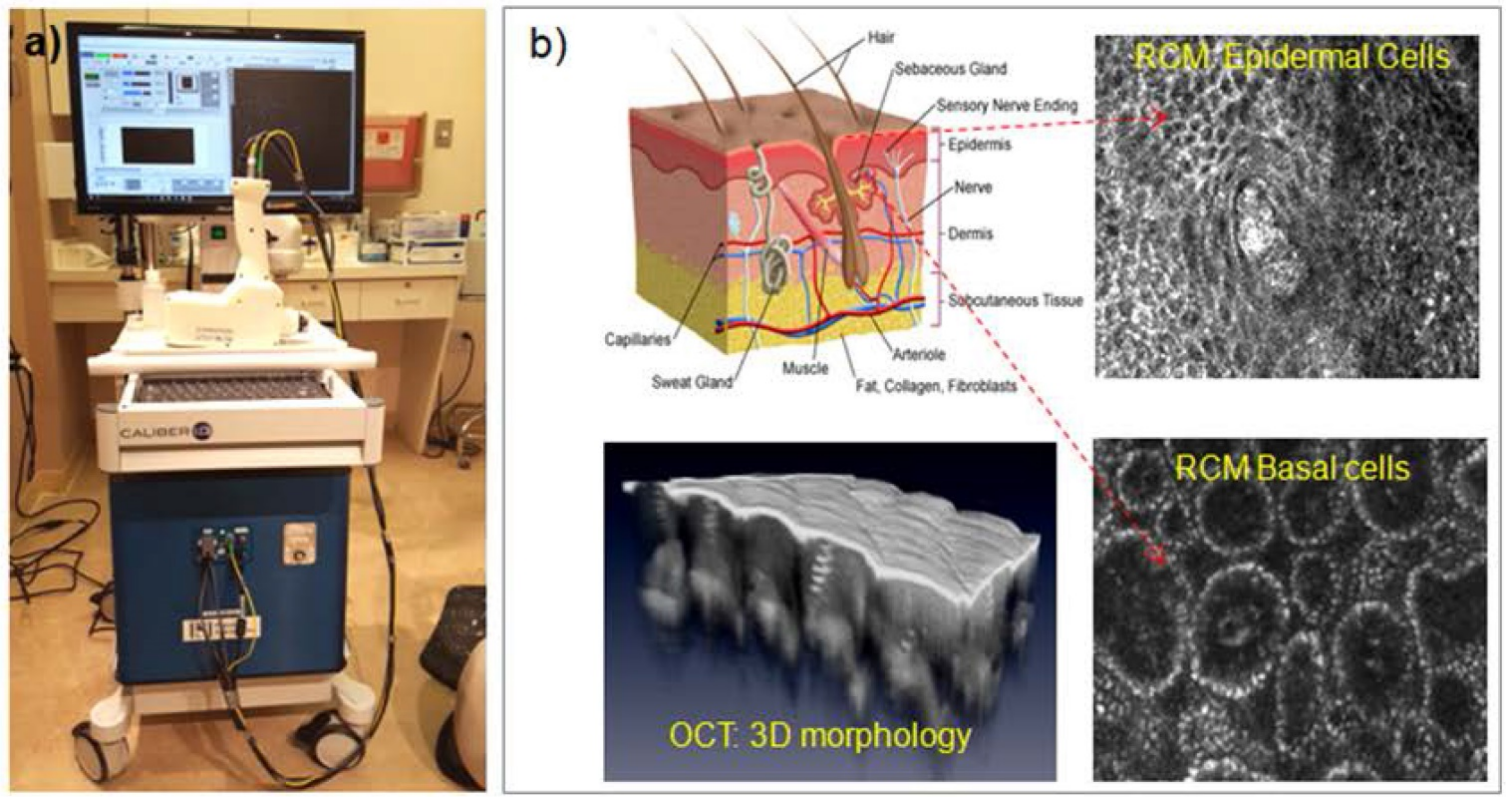
(**a**) Photograph of the RCM-OCT instrument and hand-held probe; (**b**) Representative RCM and OCT images of the healthy skin.

The integrated RCM-OCT imaging instrument was designed and engineered at Physical Sciences Inc. and clinically tested at Memorial Sloan Kettering Cancer Center (MSKCC). A photograph of the RCM/OCT instrument and its imaging capabilities are shown in **Figure 1** 27. As observed, a hand-held probe is attached to an instrumentation wheeled cart through electrical and fiber optics cables. This probe, weighting approximately 3 lb, enables rapid collection of spatially co-registered RCM-OCT images, and thus informed therapy decision making. The contact with the patient skin is made through a sterilzable plastic window, which is gently pressed against skin. To avoid secular reflection from the skin, an index matching microscopy oil is used.

In a pilot clinical study on 85 patients, 60 with lesions that were clinically suspicious (but not biopsied) for BCCs (n=60) and 25 with biopsy-proven BCCs, we correlated features of BCCs seen on RCM and OCT images with those seen in histopathology, calculated diagnostic accuracy and correlated depth predicted by OCT with histopathologically measured depth 28. Histology data were analyzed by a clinical histopathologist from MSKCC, while OCT/RCM data were analyzed by four readers, trained to interpret these images. The RCM/OCT readers were blind to histology results, as the RCM/OCT data was analyzed immediately after acquisition, while histology preparation and analysis took several more days.

RCM/OCT features of interest for BCC diagnosis used by other investigators in previous independent RCM and OCT studies were analyzed. These features are presented in **Table 1.**

## Results and Discussion

**Figure 2:**
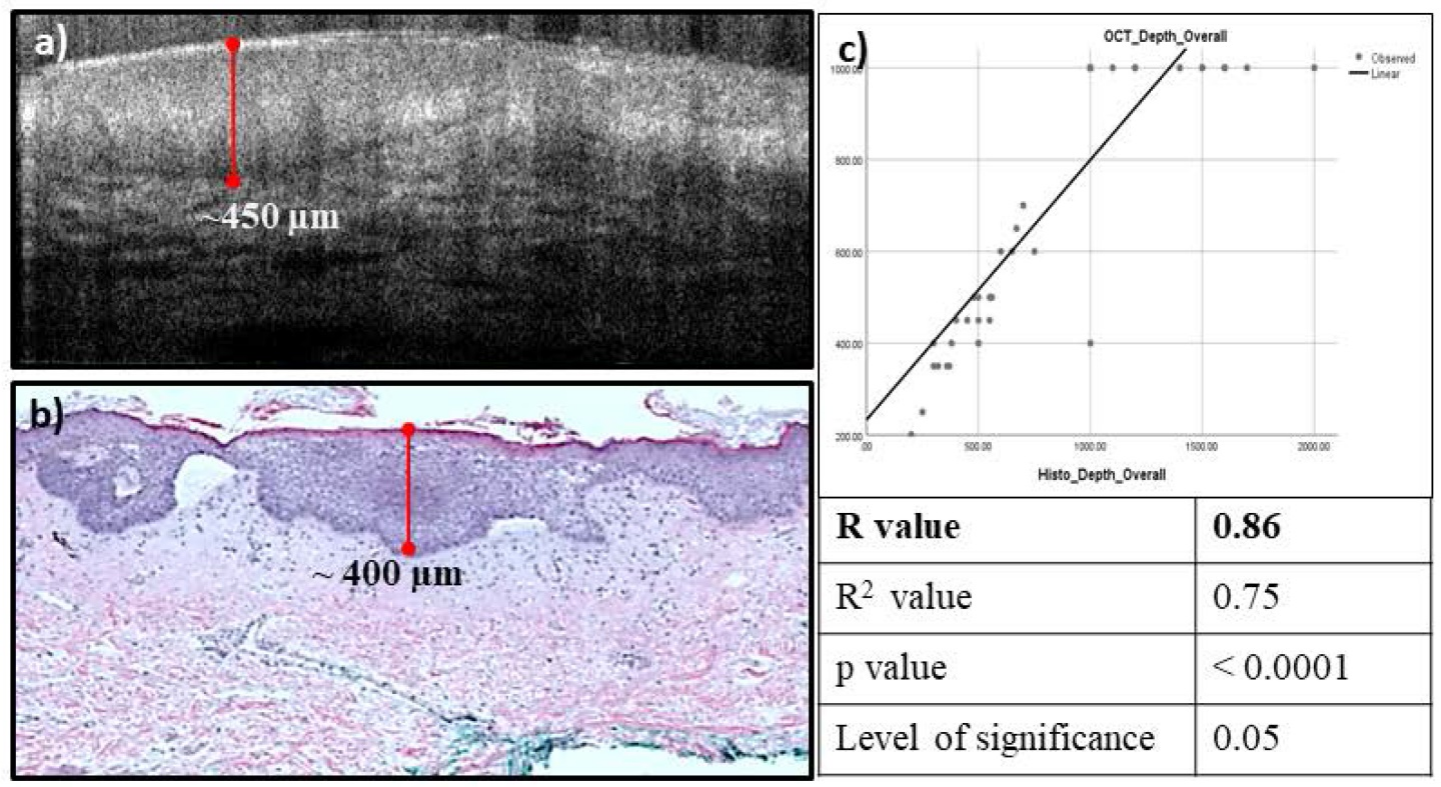
High Correlation observed between the estimated OCT depth (**a**), and the measured histopathology depth (**b**), the correlation coefficient was found to be 0.86 (**c**).

The main RCM/OCT features used in BCC diagnosis, such as small tumors within extending from the base of the epidermis; small and large tumor nests; tumor in dermis; dark silhouettes; dilated blood vessels; horn cysts; and bright peritumoral stroma were analyzed. Additional features such as necrosis and intratumoral mucin pools were correlated on OCT and histology. Higher sensitivity and negative predictive value (100%) and comparable specificity (48% vs 56% on RCM) and positive predictive value (82.19 vs 84.59 % on RCM) were observed for integrated RCM/OCT imaging for diagnosis of all lesions (n=85). Relatively higher specificity (94.1%) and positive predictive value (75%) were observed in the clinically suspicious lesions (n=60). High correlation was observed (R=0.86) between the OCT-predicted depth and histopathologically measured depth (see example in **Figure 2**).

**Figure 3:**
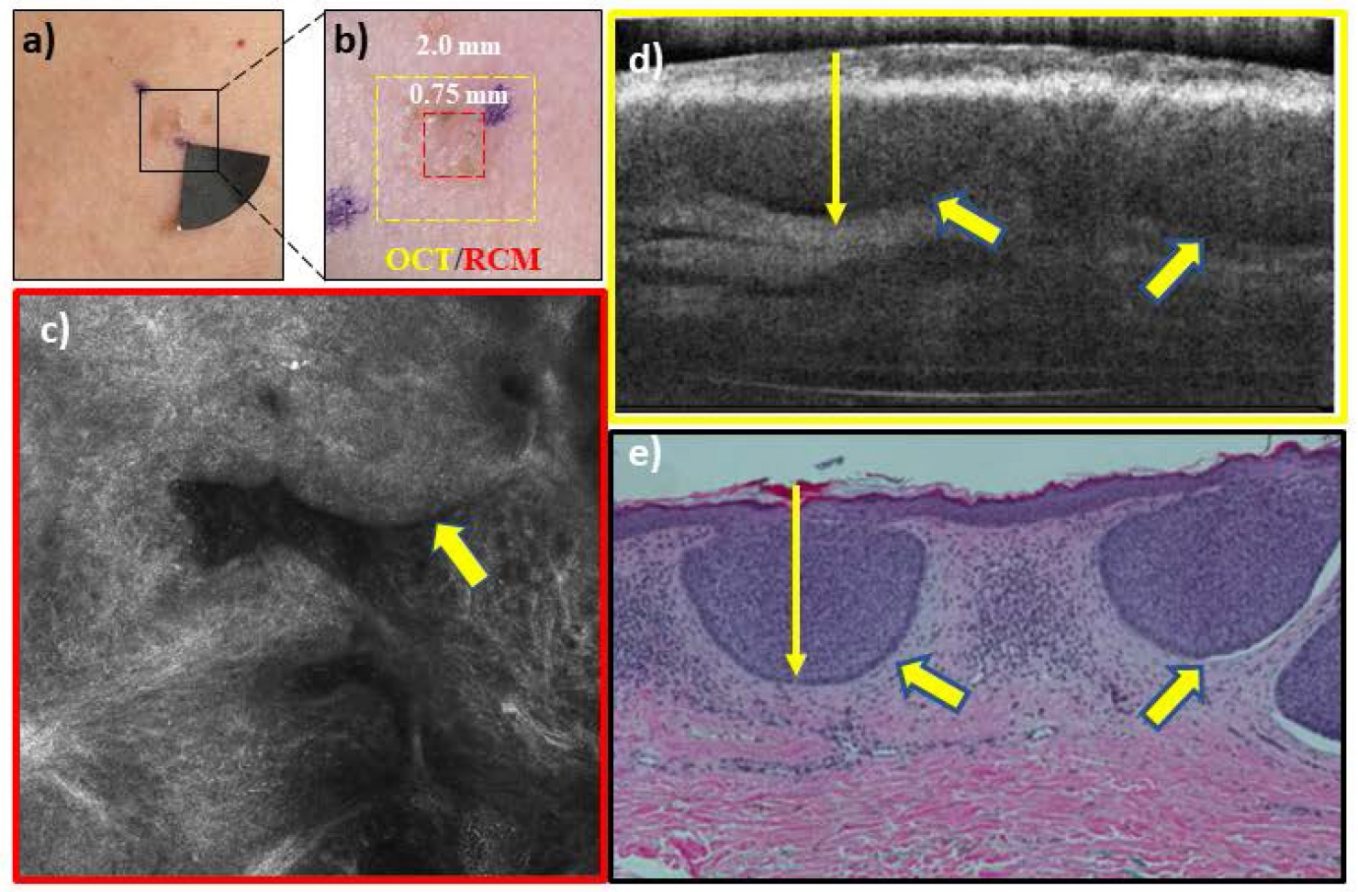
RCM-OCT imaging of a lesion on the chest of a 42 year woman which was clinically (**a**), and dermoscopically (**b**) suspicious for a basal cell carcinoma (BCC). The RCM image (**c**) shows tumor nests (yellow arrow) extending from the epidermis into superficial dermis with peripheral palisading and dark peritumoral rim- features diagnostic for BCC. The OCT image (**d**) shows a hyporeflective tumor nest (yellow arrow) with a dark peritumoral rim within the same spatial area. Based on the overall imaging, the lesion was called a superficial BCC with a depth of ~400 µm which can be treated with noninvasive approaches or superficial shave biopsy. Histopathology (e) confirmed the presence of superficial BCC with a depth of ~350 µm.

**Figure 4:**
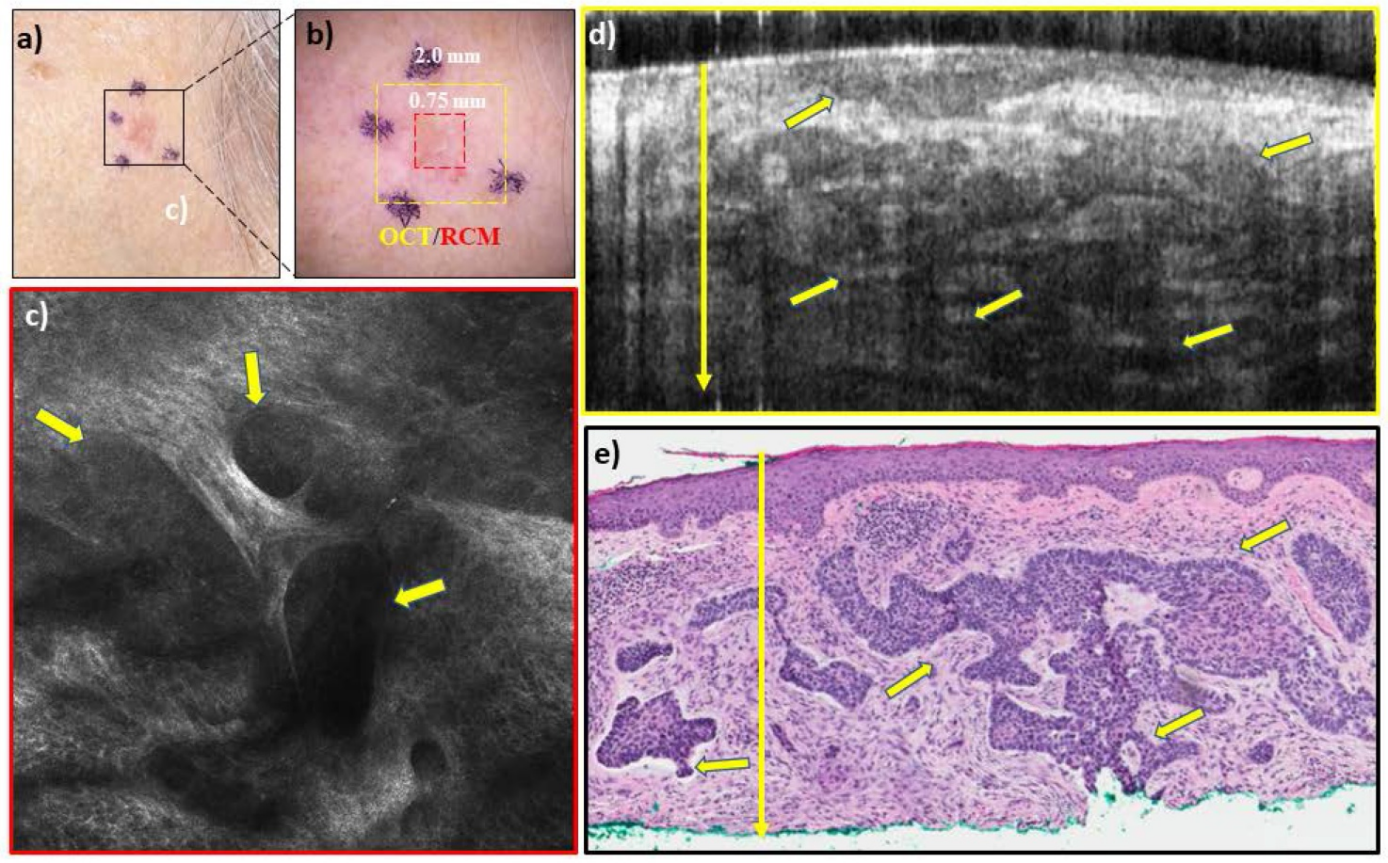
RCM-OCT imaging of a lesion on left cheek of an 83 year old woman which was clinically (**a**), and dermoscopically (**b**) suspected to be a basal cell carcinoma (BCC). The RCM images (**c**) shows large and small tumor nests (yellow arrows) in upper dermis with peripheral palisading and dark peritumoral rim. The OCT image (**d**) shows several elongated hyporeflective tumor nests even in the deeper dermis. Based on the overall imaging, the lesion was called a noduloinfiltrative BCC with a depth of >1000 um which is treated with surgical options. Histopathology (**e**) confirmed the presence of nodulo-infiltrative BCC with a depth of >1000 µm.

In addition to diagnosis and depth assessment, we are investigating the complementary capabilities of integrated RCM-OCT imaging for identifying aggressive and non-aggressive subtypes. This is crucial, as management of BCCs depends on both depth and subtype. Non-aggressive subtypes (**Figure 3),** which make up about 60% of all BCCs, can be treated with non-surgical approaches, while aggressive subtypes (**Figure 4**) require surgical management and depth assessment. Our latest studies, in progress, show preliminary promise of RCM-OCT for stratifying aggressive and non-aggressive BCCs. The additional capability for stratifying subtypes may help in lesion triage into surgical and non-surgical treatments. Additionally, RCM/OCT may also enable non-invasive monitoring of non-surgical treatments (in the absence of conventional pathology) to ensure tumor clearance. RCM/OCT imaging may be prospectively used to comprehensively diagnose suspicious lesions, determine aggressiveness and depths, triage for treatment and help in monitoring response to treatment.

## Conclusion

In conclusion, our results suggest that an integrated RCM/OCT noninvasive imaging approach shows promise for guiding BCC diagnosis and even more important for providing reliable margin and depth assessment. Recent work also indicates RCM/OCT promise for determining BCC subtype. However, larger prospective clinical trials are needed to confirm our initial findings and determine whether RCM/OCT imaging may potentially serve as an adjunct to biopsy and histopathology, and thus to guide therapy.

## Figures and Tables

**Table 1:** Integrated RCM-OCT features for BCC diagnosis

RCM Features	OCT Features
Epidermal streaming	Hyporeflective or gray structures attached to the dermal-epidermal junction (DEJ)
Tumor nests	Disruption of the DEJ
Cordlike structures	Hyporeflective or gray ovoid structures in the dermis
Nuclear palisading at tumor edge	Dark peritumoral rim (clefting)
Dark peritumoral rim (clefting)	Hyperreflective peritumoral stroma
Stroma with plump cells and bright dots	Hypo- and hyper-reflective streaks in dermis
Horizontal vessels	Branched vessels
